# Supplementary material for: Identification of Freezing-Responsive microRNAs and Their Targets in Chinese Jujube by Small RNA and Degradome Sequencing
Source: Int J Mol Sci. 2026 Sep 4;27(17):7912. doi: 10.3390/ijms27177912 (PMC13566890; doi:10.3390/ijms27177912)
Supplement: Supplementary file 1 [file ijms-27-07912-s001.zip › Supplementary Material S2/novel_miR_120.pdf]

Provisional ID : novel_miR_120
Score total : 432.7
Score for star read(s) : 3.9
Score for read counts : 425.8
Score for mfe : 1.4
Score for randfold : 1.6
Score for cons. seed :
Total read count : 847
Mature read count : 822
Loop read count : 0
Star read count : 25

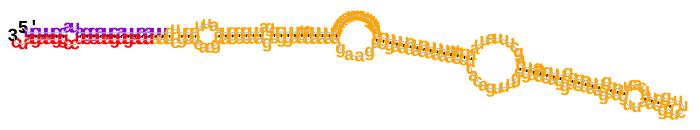

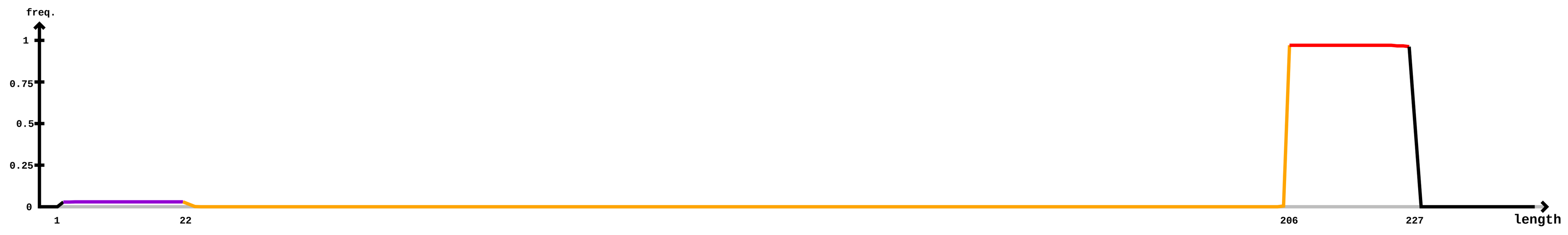


```
    Star                                                                                                                                                                                                                        Mature
5'- gaucuuccauaggaucauuaauuguugauuaauggguuaggcuuguauuuuggaaaauuuauggguuagguuuauauuuuuggaauuauuucgaguugaguugggauuggauucacuuuggcuucuggcaauuguugaaucuggacuagcguuugacacuccaaaauauaagcugaagaaaauuaggcgaacccaugcacucgcuaauuagugauccuccggaagaucauaauccaguuuggaaccaauu  -3'  obs
    gaucuuccauaggaucauuaauuguugauuaauggguuaggcuuguauuuuggaaaauuuauggguuagguuuauauuuuuggaauuauuucgaguugaguugggauuggauucacuuuggcuucuggcaauuguugaaucuggacuagcguuugacacuccaaaauauaagcugaagaaaauuaggcgaacccaugcacucgcuaauuagugauccuccggaagaucauaauccaguuuggaaccaauu        exp
    (((((((..(((((((((((((.(((...(((((((..(((((.(((((................((((((((((((.(((..........(((.((((.(((((.(((.....(((.(....).)))..))).))))).)))))))........)))))))))))))))....))))))))))).)))))))....))).))))))))))))))..))))))))....((((...)))).......  reads  mm  sample
    ..ucuuccauaggaucauuaauu...................................................................................................................................................................................................................................  1  0  JT-2
    ..ucuuccauaggaucauuaauu...................................................................................................................................................................................................................................  4  0  JCK-2
    ..ucuuccauaggaucauuaauu...................................................................................................................................................................................................................................  10  0  JCK-3
    ..ucuuccauaggaucauuaauu...................................................................................................................................................................................................................................  2  0  LT-3
    ..ucuuccauaggaucauuaauu...................................................................................................................................................................................................................................  1  0  JT-3
    ..ucuuccauaggaucauuaauu...................................................................................................................................................................................................................................  6  0  JCK-1
    ....uuccauaggaucauuaauugu.................................................................................................................................................................................................................................  1  0  JCK-3
    ..............................................................................................................................................................................................................auuagugauccuccggaag.........................  1  0  JCK-2
    ..............................................................................................................................................................................................................Cuuagugauccuccggaagauc......................  2  1  JCK-1
    ..............................................................................................................................................................................................................Cuuagugauccuccggaagauc......................  1  1  JCK-3
    ...............................................................................................................................................................................................................uuagugauccuccggaag.........................  1  0  JCK-1
    ...............................................................................................................................................................................................................uuagugauccuccggaag.........................  1  0  JCK-2
    ...............................................................................................................................................................................................................uuagugauccuccggaagUu.......................  2  1  LT-2
    ...............................................................................................................................................................................................................uuagugauccuccggaagUu.......................  1  1  JCK-1
    ...............................................................................................................................................................................................................uuagugauccuccggaagau.......................  1  0  JCK-3
    ...............................................................................................................................................................................................................uuagugauUcuccggaagauc......................  1  1  JCK-2
    ...............................................................................................................................................................................................................uuagugaucAuccggaagauc......................  1  1  JCK-2
    ...............................................................................................................................................................................................................uuCgugauccuccggaagauc......................  1  1  JT-2
    ...............................................................................................................................................................................................................uuagugauccuccggaagauU......................  1  1  LT-3
    ...............................................................................................................................................................................................................uNagugauccuccggaagauc......................  1  1  JCK-2
    ...............................................................................................................................................................................................................uuagugauccuccggaagauc......................  8  0  LT-1
    ...............................................................................................................................................................................................................uuagugauccuccggaagauc......................  12  0  LT-2
    ...............................................................................................................................................................................................................uuagugauccuccggaagCuc......................  1  1  JCK-1
    ...............................................................................................................................................................................................................uuCgugauccuccggaagauc......................  1  1  JCK-2
    ...............................................................................................................................................................................................................uuagugauccuccggaagauc......................  326  0  JCK-2
    ...............................................................................................................................................................................................................uuagugauccuccggaagauU......................  1  1  JCK-2
    ...............................................................................................................................................................................................................uuagugauccuccggaagauU......................  2  1  JCK-3
    ...............................................................................................................................................................................................................uuagugauccuccggaagauc......................  220  0  JCK-3
    ...............................................................................................................................................................................................................uuagugauccuccggaagauc......................  6  0  LT-3
    ...............................................................................................................................................................................................................uuagugauccuccggaagauc......................  23  0  JT-3
    ...............................................................................................................................................................................................................uuagugauccuccggaagauc......................  177  0  JCK-1
    ...............................................................................................................................................................................................................uuagugauccuccggaagauc......................  8  0  LCK-3
    ...............................................................................................................................................................................................................uuagugauccuccggaagauc......................  21  0  JT-2
```

Star                                                                                                                                                                                                                 Mature

gaucuuccauaggaucauuaauuguugauuaauggguuaggcuuguauuuuggaaaauuuauggguuagguuuauauuuuuggaauuauuucgaguugaguugggauuggauucacuuuggcuucuggcaauuguugaaucuggacuagcguuugacacuccaaaauauaagcugaagaaaauuaggcgaacccaugcacucgcuaauuagugauccuccggaagaucauaauccaguuuggaaccaauu

...............................................................................................................................................................................................................uuagugauccuccggaCgauc...................... 1 1 JCK-2
